# Solitary Intracranial Plasmacytoma of the Brain Treated With Primary Radiation Therapy

**DOI:** 10.7759/cureus.49798

**Published:** 2023-12-01

**Authors:** Abigail L Kohut-Jackson, Sagun D Goyal, Danielle H Carpenter, Jeevin Shahi

**Affiliations:** 1 Department of Radiation Oncology, Saint Louis University School of Medicine, St. Louis, USA; 2 Department of Internal Medicine, Division of Hematology, Oncology, Bone Marrow Transplant and Cellular Therapy, Saint Louis University, St. Louis, USA; 3 Department of Pathology, Division of Anatomic Pathology, Saint Louis University, St. Louis, USA; 4 Department of Radiation Oncology, Saint Louis University, St. Louis, USA

**Keywords:** primary brain tumors, definitive local radiation therapy, solitary extramedullary plasmacytoma (sep), intracranial plasmacytoma, volumetric-modulated arc therapy

## Abstract

We present a rare case of a solitary intracranial plasmacytoma of the brain parenchyma in a 49-year-old female who presented with neck pain/headache, paresthesias, and auditory hallucinations. A workup revealed a solitary left parietal lobe brain lesion and a biopsy demonstrated a plasma cell infiltrate consistent with an extramedullary plasmacytoma. A complete workup for multiple myeloma was negative. As opposed to surgical resection and adjuvant radiation therapy (RT), as described in prior case reports in the literature, this patient was managed with definitive local RT alone to 50 Gy in 25 fractions. Six months following primary RT completion, the patient’s presenting symptoms completely resolved and follow-up imaging revealed regression of the primary tumor. To our knowledge, this is the first reported case of a solitary extramedullary plasmacytoma of the brain treated with localized definitive RT alone.

## Introduction

A solitary plasmacytoma (SP) is a rare, localized neoplasm consisting of monoclonal plasma cells without evidence of systemic disease. These tumors are categorized as either solitary plasmacytoma of bone (SPB) or solitary extramedullary plasmacytoma (SEP), and account for approximately 4-5% and 2-3% of all plasma cell dyscrasias, respectively [1,2]. By definition, an SP does not meet the diagnostic criteria for multiple myeloma (MM) and is typically managed with definitive local treatment, either primary radiation therapy (RT) and/or surgery [3,4]. Approximately 50% of SPB and 30% of SEP will progress to MM within 10 years of diagnosis; therefore, it is important to routinely monitor these patients for progression to MM following initial treatment [5]. Anatomically, SPB most commonly involves the vertebral column, and patients present with pain and/or spinal cord/nerve root compression [5]. SEP can involve any organ system, but is generally found in the head/neck, lungs, and gastrointestinal (GI) tract, and presenting symptoms may include headaches, nasal obstruction, shortness of breath, or GI complaints [3]. Several case reports have also noted SEP of the central nervous system (CNS), connective tissue, thyroid, breast, testis, and lymph nodes [6]. In the setting of an unknown primary malignancy, it is important to consider the diagnosis of an SP since these tumors can occur anywhere with varying clinical presentations.

In exceedingly rare cases, an SEP may originate within the brain parenchyma and is referred to as a solitary intracranial plasmacytoma (SIP) of the brain. Unlike skull-based or extramedullary lesions which compress or secondarily invade the brain, SIP of the brain originates within the brain parenchyma and is an exceedingly rare diagnosis with an uncertain clinical course. The exact pathophysiology of primary brain involvement is unknown, but it has been suggested that neoplastic B-cell transformation may occur ubiquitously, even within the CNS [7]. Accurate diagnosis of SIP of the brain may also be challenging, owing to its rarity and lack of radiographic and clinical diagnostic criteria. Here, we describe a case of a SIP of the brain, which to our knowledge, is the first reported case to be treated with localized definitive RT alone. 

## Case presentation

**Figure 1:**
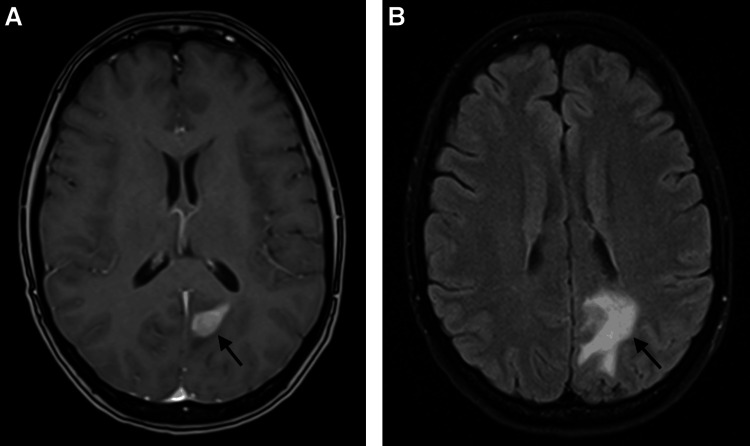
Pre-biopsy and pre-treatment T1 with contrast (A) and T2-FLAIR (B) axial MRI. Pre-biopsy and pre-treatment images demonstrating the left parieto-occipital solitary intracranial plasmacytoma with homogeneous contrast enhancement (A; arrow indicates solitary intracranial plasmacytoma). Surrounding T2 FLAIR hyperintensity is suggestive of vasogenic edema (B; arrow indicates vasogenic edema).

A 49-year-old female initially presented to her primary care provider with generalized neck pain, a 4-month history of intermittent headaches, tingling and dizziness while laying on her side, and waking from sleep with a “knocking noise” in her head. A review of systems and past medical history were otherwise unremarkable. Brain magnetic resonance imaging (MRI) demonstrated a 1.5 cm contrast-enhancing lesion in the deep left parietal lobe with surrounding vasogenic edema (Figure 1). The patient was evaluated by neurosurgery and found to have a normal neurological examination. Brain MRI was repeated and again revealed a 1.2 x 2.1 cm enhancing mass in the left medial parietal lobe. Due to the lesion’s homogenous enhancement and surrounding T2-FLAIR hyperintensity, the differential diagnoses included CNS lymphoma, glioma, and metastasis. Staging computed tomography (CT) imaging of the chest, abdomen, and pelvis was negative. Laboratory testing, including complete blood count (CBC), complete metabolic panel (CMP), and lumbar puncture (LP) for malignant cells was unremarkable. Given the negative workup and indeterminate brain imaging, a brain biopsy was recommended to achieve a pathological diagnosis.

**Figure 2:**
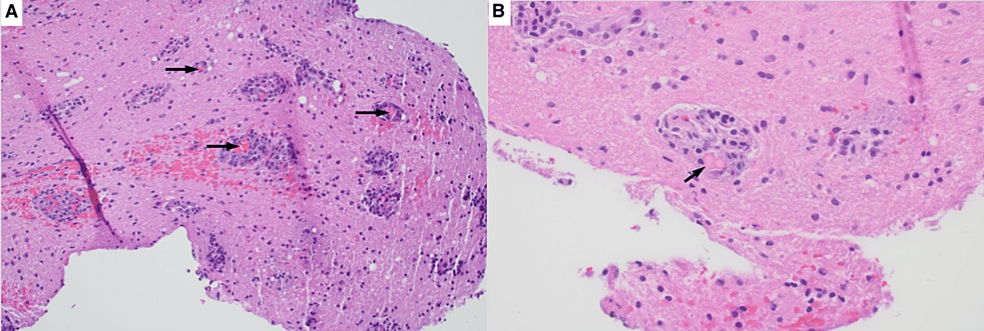
Stereotactic brain biopsy specimen. Hematoxylin and eosin stain of the left parieto-occipital stereotactic brain biopsy specimen. A: Light microscopy image (original magnification x200) showing parenchymal involvement by plasma cells predominantly in the perivascular space (arrows indicate blood vessels surrounded by plasma cells). B: Light microscopy image (original magnification x400) showing a perivascular cluster of plasma cells with rare Russell bodies (arrows indicate Russel bodies).

Left parietal stereotactic brain biopsy was uncomplicated, and pathology revealed brain parenchymal involvement by plasma cells (positive for CD 79a, CD138, MUM-1, and cytoplasmic Lambda) predominantly within the perivascular space, with rare Russell bodies noted (Figure 2). Flow cytometry revealed a monoclonal plasma cell population positive for cytoplasmic Lambda, CD19, CD38, and CD56 (partial positive). These findings were consistent with a plasma cell neoplasm, either CNS plasmacytoma or secondary myelomatous involvement of the CNS. Further workup for MM was undertaken. A whole-body positron emission tomography (PET) CT and MRI spine did not reveal any suspicious bone or soft tissue lesions. Bone marrow biopsy (BMB) did not identify a clonal plasma cell population and fluorescence in situ hybridization (FISH) analysis of the BMB sample using MM panel probes was negative. Serum LDH, beta-2 microglobulin, urine/serum protein electrophoresis, urine/serum immunoglobulins, and serum-free light chains were within normal limits. Given the negative MM workup, the patient was diagnosed with a SIP of the brain and, after multidisciplinary consensus, was referred to radiation oncology for management with primary RT.

**Figure 3:**
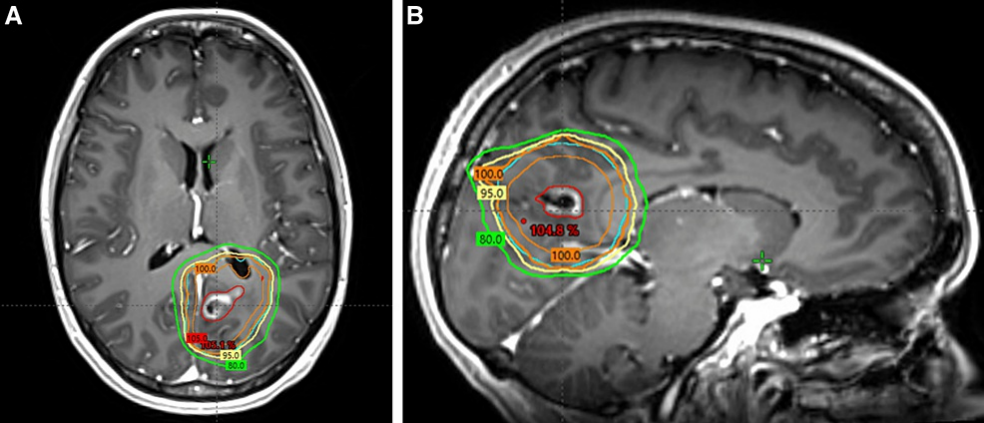
Radiation dose distribution. Radiation dose distribution demonstrating the gross tumor volume (red), clinical target volume (orange), and planning target volume (cyan). The 100% (orange), 95% (yellow), and 80% (green) isodose lines are shown on the pre-treatment axial (A) and sagittal (B) T1 MRI with contrast.

**Figure 4:**
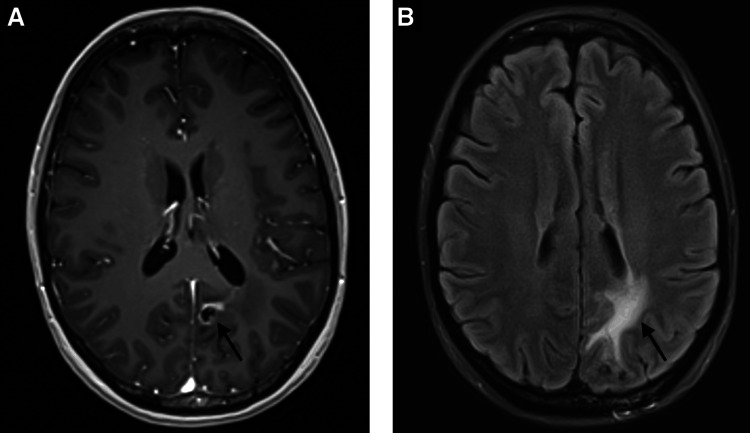
One-year post-treatment T1 with contrast (A) and T2-FLAIR (B) axial MRI sequences. Post-treatment imaging demonstrating minimal residual enhancement and no evidence of progressive disease (A; arrow indicates post-radiation changes). Surrounding T2 FLAIR hyperintensity is reduced and consistent with decreased vasogenic edema and treatment effect secondary to RT (B; arrow indicates reduced vasogenic edema).

The patient was treated with definitive external beam RT to a total dose of 50 Gy delivered in 25 fractions using a volumetric modulated arc therapy (VMAT) technique. The contrast-enhancing tumor was delineated as the gross tumor volume (GTV) and a 10 mm uniform expansion was applied as the clinical target volume (CTV). The CTV was cropped from the falx and ventricles. Areas of T2 FLAIR hyperintensity surrounding the tumor were encompassed within the CTV margin. Immobilization was with a standard thermoplastic mask and a 3 mm planning target volume (PTV) was utilized per institutional policy (Figure 3). Except for mild fatigue, the patient did not experience any other acute or late effects (to date) related to RT. Post-radiation MRI obtained 2 months after treatment demonstrated a reduction in the size of the enhancing mass with minimal residual post-biopsy contrast enhancement. Follow-up brain MRIs up to 12 months post-treatment continue to show stable post-treatment and biopsy changes, improvement in surrounding vasogenic edema, and no evidence of intracranial progression (Figure 4). The patient is followed by a radiation oncologist and hematologist-oncologist every 3-6 months and has no evidence of MM on surveillance laboratory investigations. Clinically, her presenting symptoms completely resolved 6 months after the completion of RT, and she is without any limitations in her performance status.

## Discussion

The literature describing SIP of the brain is mostly comprised of case reports, owing to the rarity of this diagnosis. In fact, to our knowledge, only 12 case reports have documented an SIP originating within the brain parenchyma (Table 1) [8-18]. In these cases, surgical resection followed by adjuvant RT has been the mainstay of treatment. For example, most recently, Yanagihara et al. (2020) described a case of a 33-year-old female with a primary cerebellar SIP, treated with subtotal resection and 50 Gy of localized post-operative RT [17]. This patient was followed for 16 months post-surgery without evidence of tumor recurrence or the development of MM. Another recent case report presented by Kumar et al. (2019) highlighted a primary occipital SIP in a 52-year-old female that was treated with gross total excision and decompressive craniectomy followed by 39 Gy in 13 fractions of palliative whole brain RT [16]. At 6 months postoperatively, the plasmacytoma decreased in size without evidence of disease recurrence [16]. As an alternative to surgical resection, definitive RT alone can be considered for SP (both SBP and SEP). Per the National Comprehensive Cancer Network (NCCN) guidelines, definitive RT doses of 40-50 Gy in 1.8-2.0 Gy per fraction are recommended (along with surgery only if necessary) [4]. Although these guidelines for RT dosing are intended to generally inform SP treatment and are not specific for SIP of the brain, we successfully treated our patient with 50 Gy in 25 fractions, which was well tolerated and resulted in regression of the primary tumor. To our knowledge, the present case represents the first successful treatment of SIP of the brain with primary local RT. We do note a case of a plasmacytoma of the left insular lobe and thalamus that was not surgically resected, but rather successfully treated with whole brain RT and combined dexamethasone [15]. A non-surgical approach was undertaken in this case due to the extensive brain infiltration of the lesion. Whole brain RT consisting of 36 Gy in 20 fractions and a boost to the primary lesion of 9 Gy in 5 fractions resulted in tumor control, and the patient remained without disease progression for the four years of follow-up reported [15]. While evidence is limited due to the rarity of SIP, the present case as well as the prior case treated with whole brain RT and dexamethasone without surgical resection [15] have been successful, and appear to have comparable efficacy to cases in which surgery was utilized.

**Table 1:** Known Cases of Solitary Plasmacytomas of the Brain

Case	Author	Age	Sex	Anatomic Location	Operation?	Radiation Therapy?	Outcome
1	French 1947 [8]	42	F	Hypothalamus	Biopsy	-	Died of ventriculitis 2 months post-biopsy.
2	Adams RD et al., 1973 [9]	72	M	Diffuse cerebral	-	-	Died in hospital at 39 days post admission for neurological impairment.
3	Goriachkina 1979 [10]	18	F	Hypothalamus	Unknown	Unknown	Unknown
4	Kanie et al., 1981 [11]	45	M	Parietal	Subtotal resection	Local RT (49 Gy)	Resolution of some symptoms at 12 months post-operatively.
5	Krumholz et al., 1982 [12]	42	M	Parietooccipital	Gross total resection	Whole brain RT (34 Gy) and tumor bed boost (6 Gy)	Residual hemianopia, otherwise asymptomatic at 8 years post-operatively.
6	Wisniewski et al., 1990 [13]	56	M	Occipital	Gross total resection	Unknown technique, 50.4 Gy	Unknown
7	Mihaljevic et al., 1996 [14]	42	F	Temporal	Gross total resection	Whole brain RT (40 Gy) and tumor bed boost (20 Gy)	Asymptomatic, 7.5 years post-operatively.
8	Mihalhevic et al., 1996 [14]	25	F	Parietal	Gross total resection	Whole brain RT (40 Gy) and tumor bed boost (20 Gy)	Asymptomatic, 5.5 years post-operatively.
9	Ferrari et al 2012., [15]	50	F	Insula and thalamus	Biopsy	Whole brain RT (36 Gy) and tumor bed boost (9 Gy)	Stable with mild cognitive impairment at 48 months post-biopsy.
10	Kumar et al 2019., [16]	52	F	Occipital	Gross total resection	Whole brain RT (39 Gy)	Decreased size and edema correction seen on MRI at 6 months post-operatively.
11	Yanagihara et al., 2020 [17]	33	F	Cerebellum	Subtotal resection	Local RT (50 Gy)	No recurrence or progression at 16 months post-operatively.
12	Nazarovs et al., 2022 [18]	30	F	Frontotemporal	-	-	Unable to treat due to comorbidities.

In our case, localized RT was chosen rather than whole-brain RT, considering the small, single tumor site as well as the higher potential risk of neurocognitive decline and neurotoxicity with whole-brain RT in a young and healthy patient [19]. Although our decision to treat with localized RT over the whole brain was intended to minimize the risk of RT to healthy brain tissue, localized RT may still have potential acute or late toxicities, which must be considered in the treatment of patients with SIP. For example, it has been noted that RT may acutely cause vasodilation and endothelial cell damage, leading to cerebral edema [20]. While localized RT may restrict cerebral edema to a smaller anatomical region, the risk for related acute increases in intracranial pressure may result in headaches, nausea, seizures, mental status changes, and/or exacerbation of symptoms from the tumor itself. The late neurocognitive effects related to whole-brain RT are well established, and localized RT techniques (such as stereotactic radiosurgery) have demonstrated superior preservation of cognitive function in the treatment of brain metastases [19,21]. It is unclear how the neurocognitive effects of RT directly compare to resection; however, potential additional complications related to surgery (bleeding, infection, stroke, cardiopulmonary failure) can be avoided with definitive RT. Any operation of the brain entails significant risk to the patient, and careful multidisciplinary discussion with the patient and other providers should be undertaken before determining whether surgery is necessary.

More commonly than SIP originating in the brain parenchyma, SIP may arise in the skull or meninges and then secondarily invade the brain. In a review of eight SIP cases, Bindal et al. (1995) reported that all tumors involved the dura mater [22]. Five of the eight cases demonstrated additional involvement of the overlying bone. In all cases, surgical resection (partial or complete) was performed and adjuvant RT between 37-50 Gy was utilized in six of the eight cases. Multiple myeloma was diagnosed at some point during the clinical course in four of the eight patients, three of whom were diagnosed after surgical resection. In our case, there was no involvement of the dura or bone on imaging or brain biopsy. The mechanism of SIP involving the brain parenchyma remains unknown; however, it has been suggested that monoclonal plasma cells in the periphery may secondarily migrate to the CNS [7]. Interestingly, many of the plasma cells in our case, histologically, were found in the perivascular space, which may support this hypothesis.

## Conclusions

To conclude, in a patient with a biopsy-proven SIP of the brain, definitive localized RT to a dose of 50 Gy in 25 fractions resulted in tumor regression. In this clinical situation, our patient had the advantage of a biopsy-determined diagnosis, and treatment with primary RT was undertaken, rather than upfront surgical resection, which is more commonly documented in the literature. A thorough workup for MM was unremarkable and no further treatment was recommended. This case represents the first known SIP of the brain to be treated with definitive localized RT alone, as may commonly be undertaken for any SP arising outside of the CNS. Primary localized RT for SIP should be considered, especially for cases in which surgical resection is high-risk. Though a rare diagnosis, this case demonstrates that SIP of the brain may be managed with primary localized RT, providing a valuable treatment option for patients when a nonsurgical approach is preferred.
